# High-Performance Cu_1.8_Se Nanosheets for Dual-Sensing: H_2_O_2_ Electrochemical Detection and SERS Substrate

**DOI:** 10.3390/nano15130998

**Published:** 2025-06-27

**Authors:** Ying-Chu Chen, Michael Chen, Yu-Kuei Hsu

**Affiliations:** 1 Department of Chemical Engineering & Biotechnology, National Taipei University of Technology, Taipei City 10608, Taiwan; 2 Department of Opto-Electronic Engineering, National Dong Hwa University, Hualien 97401, Taiwan

**Keywords:** copper selenide, nanosheet, electrochemical detection, surface-enhanced Raman scattering

## Abstract

A facile fabrication method was developed for the growth of Cu_1.8_Se nanosheets (NSs) on a Cu foil substrate, enabling dual-functionality as an electrochemical sensor for H_2_O_2_ and an active surface-enhanced Raman scattering (SERS) substrate. The process involved the preparation of Cu(OH)_2_ nanowires (NWs) via electrochemical oxidation, followed by chemical conversion to Cu_1.8_Se through a selenization process. The morphology, composition, and microstructure of the resulting Cu_1.8_Se NSs were systematically characterized using scanning electron microscopy (SEM), X-ray diffraction (XRD), and X-ray photoelectron spectroscopy (XPS). The Cu_1.8_Se NSs exhibited excellent electrocatalytic activity for H_2_O_2_ reduction, achieving a notably low detection limit of 1.25 μM and demonstrating rapid response and high sensitivity with a linear relationship in amperometric detection. Additionally, SERS experiments using Rhodamine B as a probe molecule and the Cu_1.8_Se NS/Cu foil as a substrate displayed outstanding performance, with a detection limit as low as 1 μM. The flower-like structure of the Cu_1.8_Se NSs exhibited linear dependence between analyte concentration and detection signals, along with satisfactory reproducibility in dual-sensing applications. These findings underscore the scalability and potential of this fabrication approach for advanced sensor development.

## 1. Introduction

Electrochemical sensing and surface-enhanced Raman scattering (SERS) are two powerful analytical techniques that have gained widespread attention for chemical and biological detection. Electrochemical sensors are valued for their high sensitivity, rapid response, and ease of miniaturization, making them highly suitable for real-time and in situ monitoring. SERS, on the other hand, offers ultrasensitive detection through vibrational fingerprinting of molecules, often reaching single-molecule sensitivity under optimized conditions. Integrating these complementary techniques into a dual-mode sensing platform allows for simultaneous electrochemical and spectroscopic analysis, enhancing analytical reliability, accuracy, and detection versatility. Such an integrated approach is highly desirable for next-generation sensors capable of handling complex biological and environmental samples.

Micro- and nanoscale transition-metal chalcogenides (TMCs) have garnered significant attention due to their unique chemical and physical properties, enabling diverse applications in electrochemical energy devices, optical sensors, thermoelectric systems, and memory devices [1,2,3]. Among these, nonstoichiometric copper selenides (Cu_x_Se, x = 1–2) stand out in electrochemical devices, such as water electrolyzers, batteries, and supercapacitors, owing to their exceptional electrocatalytic activity [4,5,6]. This enhanced electrochemical performance of Cu_x_Se is primarily attributed to the reduced electronegativity of anions and the high degree of covalency within the lattice, which collectively contribute to superior electrochemical tunability. Such tunability facilitates the adsorption of reactive intermediates on the Cu_x_Se surface via localized oxidation/reduction processes at the Cu active sites. Furthermore, a reduced bandgap improves the charge transport both at the Cu_x_Se–electrolyte interface and within the composite material [7]. Despite these advantages, the use of Cu_x_Se materials with novel properties for the electrochemical detection of biological materials remains largely unexplored. A straightforward, two-step method was proposed for fabricating Cu_1.8_Se nanosheets (NSs) on a Cu foil substrate for H_2_O_2_ sensing. Initially, Cu(OH)_2_ nanowires (NWs) were synthesized on the Cu foil via an electrochemical oxidation process. Subsequently, a room-temperature selenization process was carried out in an alkaline solution containing Se and NaBH_4_, yielding the final Cu_1.8_Se product, which was directly employed as an electrode for H_2_O_2_ detection. To the best of our knowledge, this is the first report demonstrating the use of Cu_1.8_Se NSs for H_2_O_2_ sensing.

Cu_1.8_Se is a self-doped material with a high hole concentration, resulting in localized surface plasmon resonances (LSPR). The coexistence of Cu(I) and Cu(II) in these materials serves as a source of optically active holes, enabling effective tuning of the optical properties via Cu-defect engineering, which can induce a blueshift or redshift in the absorption spectrum [8,9,10]. Additionally, Cu_1.8_Se exhibits remarkable potential as a surface-enhanced Raman scattering (SERS) substrate due to charge-transfer resonance between chemisorbed molecules and the Cu_1.8_Se surface. However, studies on the SERS activity of Cu_1.8_Se substrates remain limited. In this study, we demonstrate the dual functionality of Cu_1.8_Se NSs as sensitive SERS substrates and efficient electrodes for H_2_O_2_ detection. The Cu_1.8_Se NSs achieved an impressive enhancement factor of 8 × 10^6^ as a SERS substrate and a low detection limit of 1.25 × 10^−6^ M for H_2_O_2_ as an electrochemical electrode. These novel and cost-effective flower-like Cu_1.8_Se nanostructures not only serve as high-performance SERS substrates and electrochemical electrodes but also pave the way for the development of bio-related sensing devices based on hierarchical nanostructures.

## 2. Experiment Section

Sodium hydroxide (NaOH, 97%), sodium borohydride (NBH_4_, 98%), and selenium (Se, 99.999%) were obtained from Sigma-Aldrich. Copper foil and zinc foil were purchased from Shanghai Sinopharm Chemical Reagent Co., Ltd. (Shanghai, China) Hydrogen peroxide (H_2_O_2_, 30–50%) and sulfuric acid (H_2_SO_4_, 95%) were purchased from Fisher Scientific (Hampton, NH, USA). All chemicals were of analytical grade and used as received without further purification.

**Scheme 1:**
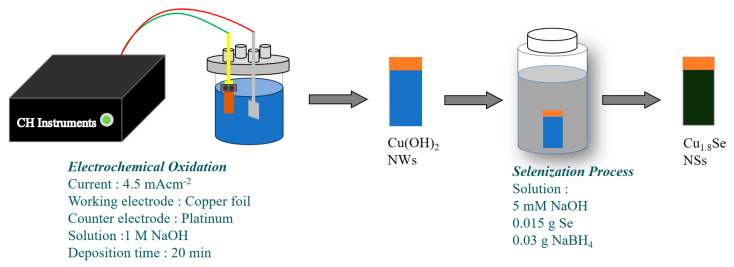
Schematic diagram of two-step process of synthesizing Cu_1.8_Se NSs.

The Cu_1.8_Se NS on the Cu foil was fabricated via a two-step process of the electrochemical oxidation process for synthesis of Cu(OH)_2_ NW and then the selenization process for the chemical composition conversion of Cu(OH)_2_ to Cu_1.8_Se, as shown in Scheme 1. In the preparation of the Cu(OH)_2_ NW, a two-electrode system of the Cu foil as a working electrode and a Pt sheet as a counter electrode in a 1 M NaOH solution was utilized. The constant current density of 4.5 mA/cm^2^ was applied for 20 min. After the reaction, the blue Cu(OH)_2_ on the Cu foil were cleaned several times by deionized water and subsequent drying in the air. For the selenization process, the Cu(OH)_2_ NW/Cu foil sample was immersed in a 40 mL aqueous solution containing 5 mM NaOH, 0.015 g of Se, and 0.03 NaBH_4_ at room temperature for 1, 6, 12, and 24 h. Finally, the sample was washed several times with deionized water and dried under atmospheric conditions. For SERS measurement, the samples were immersed in a solution of Rhodamine B at various concentrations for 1 h. Then, the samples were removed from the solution and rinsed several times with distilled water. For the electrochemical detection of H_2_O_2_, the NS samples as working electrodes were measured in a 0.1 M H_2_SO_4_ solution with a potentiostat galvanostat (CHI 6273D, CH. Instruments Inc., Bee Cave, TX, USA). A conventional three-electrode system consisting of a square platinum sheet as an auxiliary electrode and a Ag/AgCl reference electrode in a KCl solution (3 M) were implemented. All potentials reported in this article were relative to Ag/AgCl (3 M KCl, 0.197 V vs. SHE).

The morphology of the Cu_1.8_Se NS/Cu foil was analyzed by a scanning electron microscope (SEM, JEM-4000EX, JEOL (USA) Inc., Peabody, MA, USA). The structure of the samples was examined by X-ray diffraction (XRD, Rigaku D/Max-2500V X-ray Diffractometer, Tokyo, Japan) and the chemical states of the elements were determined with X-ray photoelectron spectra (XPS). Raman spectroscopy measurements were performed with a system (LabRAM HR 550, HORIBA, Irvine, CA, USA) equipped with a thermoelectrically cooled multi-channel detector (CCD) with an accuracy of 1 cm^−1^ and a 100× objective; a He-Ne laser (light power of 5 mW and wavelength of 632.8 nm) served as the excitation source. SERS data were acquired with accumulation for only 30 s for Rhodamine B on the Cu_1.8_Se NS/Cu foil samples.

## 3. Results and Discussion

### 3.1. Characteristics of Cu_1.8_Se NS/Cu Foil

**Figure 1:**
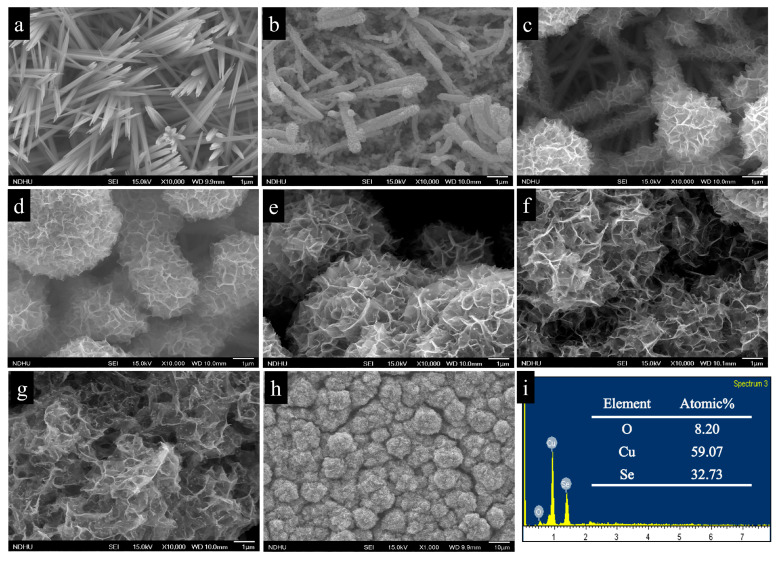
FESEM images of (**a**) Cu(OH)_2_ NWs and samples with selenization times of (**b**) 1 h, (**c**) 2 h (**d**) 4 h (**e**) 6 h, (**f**) 12 h, and (**g**) 24 h. (**h**) Low-magnification SEM of 24 h sample. (**i**) EDS of samples selenized for 24 h.

Figure 1a illustrates the morphology of Cu(OH)_2_ nanowires (NWs) directly grown on a Cu foil substrate. The one-dimensional nanostructures densely and uniformly cover the Cu foil surface, with most nanowires exhibiting a typical diameter of approximately 120 nm and a length of around 10 μm. Notably, after undergoing the selenization process for durations ranging from 1 h to 24 h, the wire-like morphology gradually disappears, giving way to sheet-like nanoflower structures, as depicted in Figure 1b–h. Each nanosheet measures several microns in width and tens of nanometers in thickness. These findings provide direct evidence for the time-dependent topotactic transformation mechanism, driven by the replacement of hydroxide with selenide ions and the accompanying reconstruction of the crystal lattice. Energy-dispersive X-ray spectroscopy (EDS) analysis (Figure 1i) reveals a Cu/Se composition ratio of approximately 1.8, along with detectable oxygen content attributed to surface oxidation from air exposure. This two-step fabrication process successfully produces dense and uniform flower-like Cu_1.8_Se nanostructures on the Cu foil substrate.

**Figure 2:**
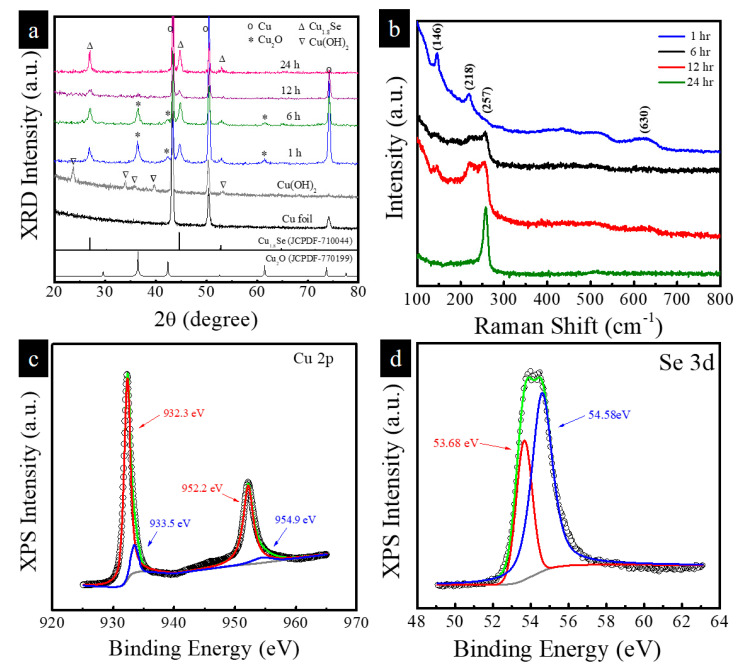
(**a**) X-ray diffraction pattern; (**b**) Raman spectra; (**c**) Cu 2p; and (**d**) Se 3d XPS of the Cu_1.8_Se NS sample.

To investigate the crystal structure of Cu_1.8_Se synthesized through this two-step process, X-ray diffraction (XRD) measurements were performed, as shown in Figure 2a. For comparison, the Cu foil substrate was also analyzed. After electrochemical oxidation, the XRD pattern of the light-blue sample reveals peaks (marked with inverted triangles) corresponding to the orthorhombic-phase Cu(OH)_2_ (JCPDS card No. 72-0140), alongside peaks (marked with circles) from the copper substrate. Following 1 h of selenization, the diffraction pattern exhibits peaks consistent with the cubic-phase Cu_1.8_Se (JCPDS card No. 71-0044, marked with triangles), along with peaks from the copper substrate and the cubic phase of Cu_2_O (JCPDS card No. 77-0199, marked with asterisks). With extended selenization time up to 24 h, the diffraction peaks corresponding to Cu_1.8_Se become more pronounced, while those associated with the Cu_2_O phase vanish. This confirms the successful conversion of Cu(OH)_2_ NWs into Cu_1.8_Se NSs through the selenization process. The possible reaction mechanisms for this transformation are as follows [11]:

8Se + NaBH_4_ + 8NaOH → 4Na_2_Se_2_ + 6H_2_O + NaBO_2_ (1)

Cu(OH)_2_ + 2OH^−^ → Cu(OH)_4_^2−^ (2)

Cu(OH)_4_^2−^ + NaBH_4_ + 4OH^−^ → Cu^+^ + 6H_2_O + NaBO_2_ (3)

1.8Cu^+^ + Na_2_Se_2_ → Cu_1.8_Se + Na_2_Se (4)

These reactions collectively enable the transformation of Cu(OH)_2_ NWs into Cu_1.8_Se NSs, resulting in the desired hierarchical nanostructures.

The structural transformation of Cu_1.8_Se NSs and the influence of the selenization treatment were further analyzed using Raman scattering spectroscopy. Figure 2b presents the Raman spectra of Cu_1.8_Se samples subjected to selenization for 1, 6, 12, and 24 h. After 1 h of selenization, the spectrum is dominated by phonon modes characteristic of the Cu_2_O phase at 146 and 218 cm^−1^ [12]. However, as the selenization time increases, these peaks progressively diminish, while new phonon frequencies characteristic of crystalline Cu_1.8_Se emerge. A prominent peak at 257 cm^−1^, corresponding to the Cu–Se vibration mode in crystalline Cu_1.8_Se, becomes evident, aligning well with reported literature values [13]. Upon extending the selenization time to 24 h, the intensity of the 257 cm^−1^ peak significantly increases, while the phonon modes associated with Cu_2_O vanish. This indicates an optimal crystallization of Cu_1.8_Se NSs, consistent with the XRD results. Additionally, the Cu 2p and Se 3d core-level spectra of the Cu_1.8_Se NS/Cu foil sample were examined via X-ray photoelectron spectroscopy (XPS), as shown in Figure 2c,d. The high-resolution Cu 2p spectrum reveals two primary peaks at binding energies of 932.3 eV and 952.2 eV, corresponding to Cu⁺ 2p_3_/_2_ and Cu⁺ 2p_1_/_2_, respectively, indicating the +1 oxidation state in Cu_1.8_Se. Weak peaks at 933.5 eV and 954.9 eV are also observed, which are attributed to the +2 oxidation state. These findings are consistent with previously reported values for mixed-valent Cu_1.8_Se materials. The Se 3d spectrum exhibits peaks at binding energies of 53.7 eV and 54.6 eV, corresponding to the Se 3d_5_/_2_ and Se 3d_3_/_2_ states of Se^2−^ in Cu_1.8_Se, respectively. These observations confirm the complete conversion of Cu(OH)_2_ to Cu_1.8_Se, corroborating the results obtained from XRD and Raman analysis.

### 3.2. Detection of H_2_O_2_ by an Electrochemical Mean

**Figure 3:**
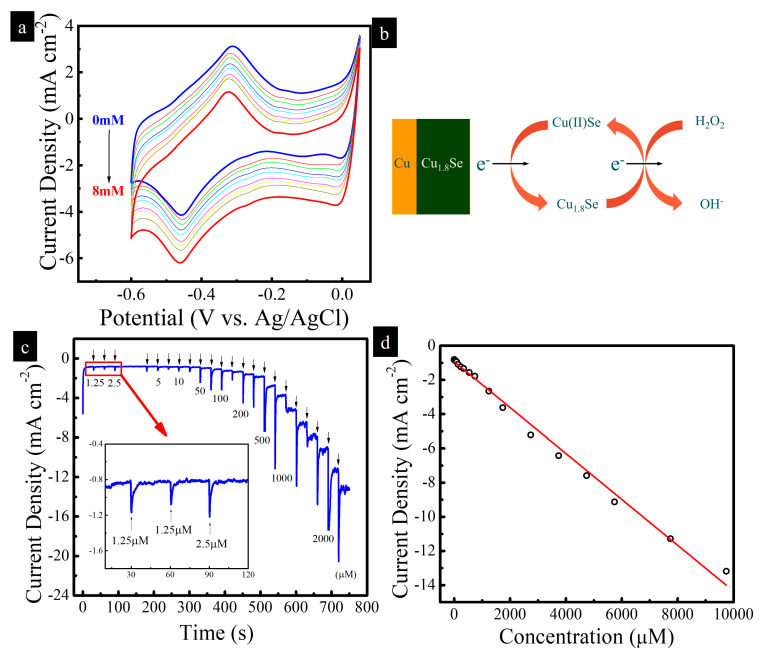
CV curves of the Cu_1.8_Se NS sample in the electrolyte (**a**) with and without varied concentrations of H_2_O_2_ and (**b**) sensing mechanism of Cu_1.8_Se. (**c**) Amperometric response at −0.2 V with increasing H_2_O_2_ concentration per 50 s for the Cu_1.8_Se NS samples; (**d**) relation between the amperometric response and H_2_O_2_ concentration.

To evaluate the electrocatalytic performance of the Cu_1.8_Se NS/Cu foil electrode after 24 h of selenization, cyclic voltammetry (CV) was conducted to study H_2_O_2_ reduction within the potential range of 0 V to −0.6 V at a scan rate of 50 mV·s^−1^. Figure 3a shows the CV curves of the Cu_1.8_Se NS electrode in an H_2_SO_4_ solution, both in the absence and presence of H_2_O_2_. In the absence of H_2_O_2_, the CV curve displays one oxidation peak and one reduction peak, which can be attributed to the redox reaction of the Cu_1.8_Se NS during the Cu(I)/Cu(II) transition, occurring at approximately −0.4 V vs. Ag/AgCl [14]. Upon the addition of H_2_O_2_, both the oxidation and reduction currents increase significantly, indicating a strong electrocatalytic response to H_2_O_2_. The mechanism for H_2_O_2_ reduction on the Cu_1.8_Se electrode can be shown in Figure 3b, and the redox cascade reaction is expressed as follows [14]:

Cu_1.8_Se → CuSe + ne^−^ (5)

H_2_O_2_ + 2e^−^ → 2OH^−^ (6)

Notably, as the H_2_O_2_ concentration increases to 8 mM, the Cu_1.8_Se NS electrode exhibits a significant increase in current density from H_2_O_2_ reduction, enabling efficient low-potential amperometric detection. These results highlight the excellent electrocatalytic activity of the Cu_1.8_Se NS, as well as the good accessibility of the nanostructures to analytes and the enhanced electron-transfer efficiency between H_2_O_2_ and the Cu_1.8_Se NS. This demonstrates the potential of the Cu_1.8_Se NS/Cu foil electrode for effective H_2_O_2_ sensing applications. The electrocatalytic activity of the Cu_1.8_Se NS electrode for H_2_O_2_ detection was evaluated using the amperometric *I*-*t* method, a widely used technique for electrochemical sensor characterization. To minimize interference from compounds such as ascorbic acid, glucose, and acetaminophen, which can contribute significantly to the anodic current and affect the accurate detection of H_2_O_2_ in practical applications, a reduction potential of approximately −0.2 V was selected. Figure 3c shows the amperometric response of the Cu_1.8_Se NS electrode to successive injections of H_2_O_2_ at varying concentrations, recorded at an applied potential of −0.2 V. The corresponding calibration curve is provided in Figure 3d. Distinct and well-defined current responses were observed for each successive addition of H_2_O_2_, demonstrating the electrode’s excellent catalytic activity. Following each injection, the catalytic reduction of H_2_O_2_ at the Cu_1.8_Se NS electrode surface rapidly reached dynamic equilibrium, producing a stable current signal within 30 s. These results confirm the stability and efficiency of the Cu_1.8_Se NS electrode’s catalytic properties. The electrode’s response to H_2_O_2_ concentration exhibits a linear range from 1.25 μM to 10 mM, with a high correlation coefficient (R^2^ = 0.990)) and a sensitivity of 1335 μA mM^−1^cm^−2^. Compared to previously reported H_2_O_2_ sensors (as detailed in Table 1) [15,16,17,18,19,20], the Cu_1.8_Se NS electrode demonstrates superior performance. This remarkable performance can be attributed to the hierarchical Cu_1.8_Se nanostructure, which provides a large surface-to-volume ratio and a high density of finely and uniformly dispersed nanosheets. These structural advantages facilitate efficient electron transfer, thereby enhancing the sensor’s electrocatalytic activity and overall performance in H_2_O_2_ detection.

**Figure 4:**
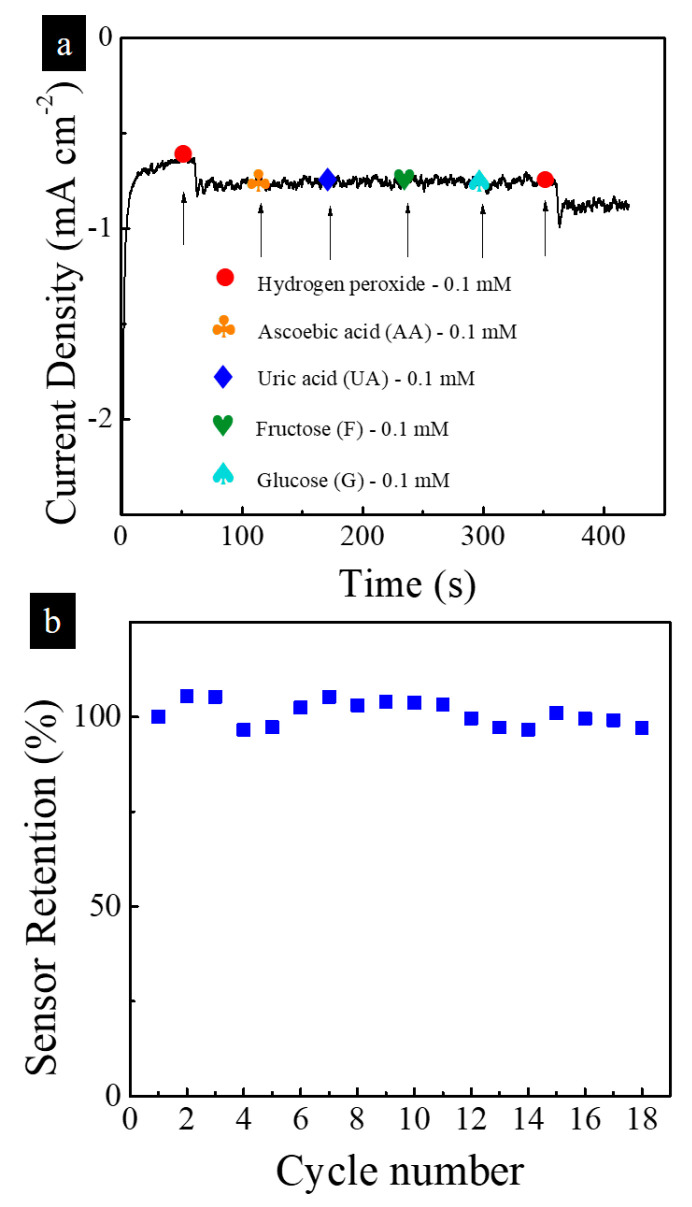
(**a**) Flow injection amperometric response to injections of interferences of fructose, ascorbic acid, uric acid, and glucose. (**b**) Stability test of Cu_1.8_Se NS samples.

To evaluate the potential impact of interfering species, the anti-interference properties of the Cu_1.8_Se NS electrode were investigated. These tests involved successive additions of common interfering compounds, including glucose, fructose, uric acid, and ascorbic acid (each at 100 μM), along with H_2_O_2_, into the electrolyte. The results, shown in Figure 4a, reveal that the Cu_1.8_Se NS electrode produces a well-defined current response for H_2_O_2_, while the current responses for the interfering species were negligible—less than 5% of that for H_2_O_2_. This demonstrates the reliable anti-interference capability of the Cu_1.8_Se NS electrode. Furthermore, the reusability of the Cu_1.8_Se NS electrode was assessed by measuring its current response to a fixed H_2_O_2_ concentration over 18 consecutive cycles, as shown in Figure 4b. The electrode maintained 89% of its initial current response after these repeated uses, highlighting its excellent durability and stability. These results confirm the Cu_1.8_Se NS electrode’s potential for practical applications, showcasing its reliable anti-interference properties and robust reusability in H_2_O_2_ sensing.

### 3.3. SERS Performances

**Figure 5:**
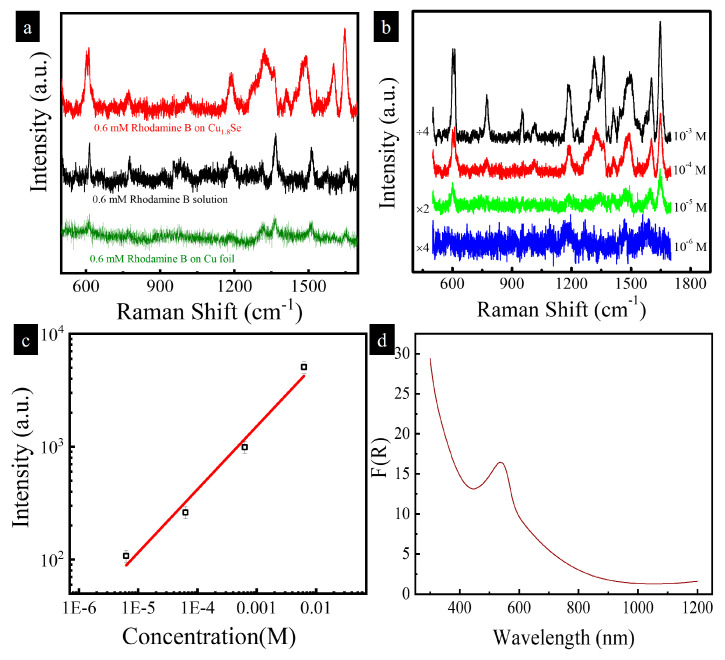
(**a**) Raman spectra of Rhodamine B adsorbed on the Cu_1.8_Se NS samples and comparison of Raman signal with a 0.6 mM Rhodamine B solution and Rhodamine B adsorbed on the Cu foil. (**b**) Raman spectra of Rhodamine B with varied concentration adsorbed on the Cu_1.8_Se NSs; (**c**) linear relation between the logarithmic intensity at 1645 cm^−1^ and concentration of Rhodamine B. (**d**) Optical absorption spectrum of the Cu_1.8_Se NS samples.

Figure 5a presents a comparison of Raman signals from a 0.6 mM Rhodamine B adsorbed on Cu_1.8_Se NS surfaces. The characteristic surface-enhanced Raman scattering (SERS) signals of Rhodamine B on the Cu_1.8_Se NS are observed at vibrational modes of 614, 1187, 1320, 1365, 1511, and 1645 cm^−1^. These vibrations correspond to ring deformation, C-H stretching, C-H in-plane bending, and aromatic C-C stretching [21]. Notably, the SERS signals from the Cu_1.8_Se NS sample are significantly stronger than those from a 0.6 mM Rhodamine B solution and a 0.6 mM Rhodamine B adsorbed on Cu foil surfaces. The SERS activity can be attributed to the LSPR effect of Cu_1.8_Se, which arises due to its high hole concentration as a p-type semiconductor [8,9,10]. In addition, the 2D nanosheet morphology enhances surface interactions and creates hot spots that contribute to the observed SERS performance. Using Rhodamine B as a test molecule, the enhancement factor (EF) of Cu_1.8_Se NS as a SERS substrate was estimated based on Equation (7) [21]:

EF = (*I*_SERS_/*I*_bulk_)(*N*_bulk_/*N*_ads_) (7)

where *I*_SERS_ and *I*_bulk_ are the Raman signal intensities at a specific vibrational wavenumber (1645 cm^−1^, chosen for this study) for Rhodamine B molecules adsorbed on the substrate and in solution, respectively. The intensity ratio was determined by measuring Rhodamine B at the same concentration, with *I*_SERS_ obtained on the Cu_1.8_Se substrate and *I*_bulk_ obtained from the same solution on a non-enhancing bare Cu foil, where no surface enhancement occurs. *N*_ads_ and *N*_bulk_ represent the number of adsorbed molecules on the Cu_1.8_Se NS surface and the number of Rhodamine B molecules in the solution within the laser spot, respectively. Following our previous method for calculating *N*_bulk_ and *N*_ads_, the ratio *N*_bulk_/*N*_ads_ was determined to be approximately 10^4^ for the same laser spot area [22]. Under identical Raman measurement conditions, the intensity ratio *I*_SERS_/*I*_bulk_ was calculated to be approximately 10^2^. Consequently, the EF was estimated to be on the order of 10^6^, which is comparable to many reports on copper-based SERS substrates (as detailed in Table 2) [23,24,25,26,27].

To further explore the SERS detection capability of the Cu_1.8_Se NS, Rhodamine B solutions of varying concentrations were tested. Figure 5b presents Raman spectra of Rhodamine B adsorbed on Cu_1.8_Se NS substrates at concentrations ranging from 1 × 10^−3^ M to 1 × 10^−6^ M. The Raman signal intensity decreases progressively as the Rhodamine B concentration is reduced, with clear detection achieved even at a concentration as low as 1 μM. This demonstrates that the novel flower-like Cu_1.8_Se NS substrate exhibits a lower detection limit compared to previously reported copper-based SERS substrates (as detailed in Table 2). Furthermore, the relationship between the intensity of the SERS spectra and the concentration of Rhodamine B using the Cu_1.8_Se NS substrate was investigated. For the four tested concentrations applied to seven experimental windows, the intensity–concentration relationship is depicted in Figure 5c. To evaluate the reproducibility of the SERS signals, measurements were performed at three randomly selected regions across three independently prepared substrates using different Rhodamine B concentrations. The intensity of the characteristic Raman peak at 1645 cm^−1^ was used for statistical analysis. The average SERS intensity was calculated, and the standard deviation and relative standard deviation (RSD) were determined. The resulting %RSD was 6.8%, indicating good signal reproducibility and uniformity across the substrate. A linear correlation was observed between the logarithm of Rhodamine B concentration and the intensity of the Raman mode at 1645 cm^−1^, as described by Equation (8):

log *I* = 4.86 + 0.56 × log *C* (8)

where *I* represents the SERS signal intensity of Rhodamine B and *C* is the concentration of Rhodamine B. To better understand the light–matter interaction and possible electromagnetic enhancement mechanism, we have now included the UV–Vis–NIR absorption spectrum of the Cu_1.8_Se nanosheet film. The spectrum shows strong and broad absorption across the visible and near-infrared regions, supporting the presence of LSPR, which is attributed to the high hole concentration in Cu_1.8_Se. This absorption behavior is consistent with literature reports on plasmonic p-type copper chalcogenides and provides further evidence that the nanosheet structure contributes to the observed SERS enhancement through light absorption and field confinement [8,9,10].

## 4. Conclusions

Two-dimensional Cu_1.8_Se nanostructures on a Cu foil substrate were successfully synthesized through a two-step process, serving as a dual-functional sensor for H_2_O_2_ detection and a highly sensitive SERS substrate. The synthesis began with the electrochemical oxidation method to produce Cu(OH)_2_ nanowires (NW), followed by selenization of the Cu(OH)_2_ NW in an alkaline solution containing NaBH_4_ and Se at room temperature for 24 h, yielding the Cu_1.8_Se NSs. The morphology, composition, and crystal structure of the Cu_1.8_Se NS were characterized using SEM, XPS, and XRD, respectively. As an electrochemical electrode for H_2_O_2_ detection, the Cu_1.8_Se NS exhibited excellent performance, including a low detection limit of 1.25 µM, outstanding stability, and effective anti-interference capability. Additionally, as a SERS substrate, the Cu_1.8_Se NS demonstrated remarkable sensitivity, enabling the detection of Rhodamine B with an enhancement factor (EF) of approximately 10^6^. In both sensing applications, the Cu_1.8_Se NS displayed a linear relationship between the concentration and detection signal, along with high reproducibility. This study highlights a simple and efficient route for fabricating Cu_1.8_Se NS structures as effective SERS substrates and promising candidates for the routine analysis of H_2_O_2_ concentration.

## Figures and Tables

**Table 1:** Comparison with the literature regarding the performance of electrochemical H_2_O_2_ sensing.

Sensor Type	Detection Limit (μM)	Linear Range (mM)	Sensitivity (μAmM^−1^cm^−2^)	Ref.
Cu_2_O/GN	20.8	0.3–7.8		[15]
ZnO_3_−CuO_7_/CPE	2.4	0.003−0.53	1.11	[16]
CuO-SiNWs/GCE	1.6	0.01–13.18	22.27	[17]
Fe_3_O_4_/GCE s	2	0.005−4.9	205	[18]
CuO nanoflowers	5	0.05-4.600	116.1	[19]
CuCo_2_O_4_	3.0	0.01–8.90	94.1	[20]
Cu_1.8_Se NS	1.25	0.00125–9.7375	1335	This work

**Table 2:** Comparison with the literature regarding the SERS performance.

SERS Substrate	Detection Limit (M)	EF	Ref.
CuSe-rGO	10^−7^	62.5	[23]
Cu/gCN	10^−9^	4.1 × 10^3^	[24]
Cu NSs		1.3 × 10^7^	[25]
AuNPs	2 × 10^−8^	38.9	[26]
CuPt alloy nanotubes	10^−6^		[27]
Cu_1.8_Se NS	10^−6^	10^6^	This work

## Data Availability

The data are contained within the article.
